# Forms of distributed leadership – a case study of six workplaces in eldercare

**DOI:** 10.1186/s12913-025-12417-1

**Published:** 2025-02-22

**Authors:** Andrea Eriksson, Monica Andersson Bäck, Magdalena Elmersjö, Gunnar Gillberg

**Affiliations:** 1 Division of Ergonomics, Department of Biomedical Engineering and Health Systems, KTH Royal Institute of Technology, Stockholm, Sweden; 2 Department of Sociology and Work Science, University of Gothenburg, Gothenburg, Sweden; 3 Department of Social Work, School of Social Sciences, Södertörn University, Stockholm, Sweden

**Keywords:** Distributed leadership, Eldercare, Case study, Realistic evaluation

## Abstract

**Background:**

The concept of distributed leadership has been addressed in previous research, but few studies link their analysis to current and comparative empirical studies on processes and conditions enabling or hindering the development of distributed leadership. This specific study aims to identify and analyze mechanisms that enable or hinder the development of distributed leadership among employees in eldercare.

**Methods:**

This is a case study based on six specific workplaces in eldercare in Sweden in different ways aiming to work toward an organization that emphasizes trust and distribution of power. A realistic evaluation framework was used to understand the different workplace program theories regarding distributed leadership. Key mechanisms and how they interact with contextual factors in each case were analyzed. Comparative analyses were performed, identifying key processes in terms of realizing distributed leadership.

**Results:**

Analyzing the program theories in the respective cases showed that they have different orientations influenced by different motives for distributed leadership, which also interact with how distributed leadership was manifested and realized. The results point specifically to the importance of the mechanism formalization processes, participatory approaches to implementation, vertical sense-making, and horizontal sense-making for the development of distributed leadership.

**Conclusions:**

The result points to that regardless of the path for achieving distributed leadership adopted by the various workplaces studied, the common denominator for those succeeding in distributing leadership is the development of a relational agency based on shared visions, a shared understanding of roles, and responsibilities, a learning approach and a dialogue-oriented relationship between management and employees. Another critical aspect is having sufficient resources to make taking on more responsibilities attractive.

**Supplementary Information:**

The online version contains supplementary material available at 10.1186/s12913-025-12417-1.

## Background

### Introduction

In recent years, the Nordic countries, known for their well-developed public care systems, have increasingly focused on innovative leadership in eldercare (e.g. [1, 2]). This shift aims to address significant challenges, such as recruitment and staff turnover, as well as tackling complex aspects of quality and safety, which are prevalent issues not only in social welfare within Nordic countries but also in eldercare across many other nations [3–5]. Distributed leadership (DL), as part of a trust-based organization, is often presented for handling these problems [1]. It is about giving the staff responsibilities and increased influence while changing the leadership in the direction of support and coaching are expected to increase satisfaction and make the workplace more attractive [5]. A systematic review also showed that interventions aiming at developing collective leadership within healthcare, including a DL largely reported positive outcomes like increased engagement and team performance [6].

The definitions of DL are not entirely straightforward, as several alternative and in some cases conflicting definitions appear in the literature. In this study, the point of departure is that DL is an alternative beyond traditional individual and hierarchical leadership which embraces increasing and distributing responsibilities and the power of decision-making to employees [7, 8]. Gronn [8, 9] describes several typical features including the concertive action and conjoint agency related DL. Concertive action concerns vertical communication and collaboration in the organization that forms a DL. First, it may concern *spontaneous collaborations*, where individuals with different skills and expertise contribute and thus gain responsibilities. Second, it may concern an *intuitive understanding* arising when members of an organization develop trust and thus deepen a working relationship with a shared understanding of responsibility taking. Last but not least, it may involve institutionalizing these relationships and activities to build collaborative structures in the organization [7, 8]. In this study, collaborative and participatory processes aiming for DL, including degrees of formalization of the collaborative structures, will be the focus of the study of the phenomenon of DL.

Conjoint agency refers, according to Gronn [8], more to the alignment and coordination of leadership actions as well as the reciprocal influences between organizational members. Curie and Lockett [7] clarify that it may include the reciprocal influence team members exercise on each other that enables individuals to take on different and complementary leadership roles forming the collective DL in the team. Martin et al. [10] discuss that conjoint agency reflects the quality of DL as cooperation in practice (i.e., whether DL is aligned or misaligned): “whether actors across the organization act with the same or different interpretations or purpose” [10]. In our study, we explore the conjoint agency by focusing on the relational and sense-making processes [11] that contribute to the extent to which extent employees experience involvement in DL practices as an outcome.

Although the concept of DL is addressed in several scientific articles [2, 3, 8, 12], only a few articles link their analysis to current and comparative empirical studies on the underlying mechanisms enabling or hindering the development of distributed leadership. Aypay and Akyüred [13] have carried out a systematic review of articles concerning DL and concluded that there has been an increasing interest in this issue since 2015. Yet, many studies concerning DL are conceptual and theoretically descriptive, why there is a demand for studies on DL in practice; staff, managers, and others who work with and try to implement DL in work groups and organizations (see also [13]). This article contributes to bridging this gap between theory and practice by analyzing six efforts to implement and apply DL in concrete, practical eldercare work.

The study is part of a larger research project focused on describing and analyzing DL, primarily in eldercare in Sweden. In this context, DL refers to transferring decision-making (e.g., increased responsibilities concerning traditional leadership tasks) and increasing participation in decision-making among operational staff [8, 14]. This specific study aims to identify and analyze mechanisms that enable or hinder the development of a DL among employees in eldercare.

### Structure of the article

The remaining parts of the article are structured as follows: First, we present the theoretical points of departures of our study, including how we define outcomes of DL, a realistic evaluation framework for analyzing implementations, critical contextual factors for DL in eldercare, critical aspects of the implementation of DL, and finally how sense-making processes may contribute to the development of DL. The method section discusses the overall approach of the research project, the specific case study design, and finally how the cases were analyzed. Consequently, in the section on results, we first present the overall outcome for each case, followed by the results of the comparative analysis of mechanisms. Finally, we discuss key findings related to critical factors in terms of developing DL.

### Outcomes of DL: involvement and participation in decision-making processes

In our study, the main outcomes are defined by the extent to which employees experience involvement and participation in DL practices, including decision-making processes. This may be expressed by leadership tasks being formally distributed to employees but may also embrace a culture of shared commitments where individual employees take informal leadership over work. DL could overall be seen as a collective activity that in different ways challenges the traditional image of leadership consisting of a single individual acting almost like a hero and carrying out (extraordinary) actions. This means that the formal and traditional leadership role is in various ways transformed into a more facilitating role offering guidance and support rather than giving instructions. As a result of this change, employee influence and responsibility naturally increase [3, 14].

Involvement in DL practices may be considered according to Arnstein’s [15] ladder of participation consisting of *different levels of participation.* The first steps in the ladder include forms of informing without any channels for feedback, as well as ways of seeking consultation and advice from, for example, operational staff while only offering them limited opportunities to have influence. More developed levels of participation, according to Arnstein’s ladder, include providing opportunities for participation through partnerships in, for example, committees, including shared decision-making processes. Within eldercare, this could mean to include staff representatives in management groups where decisions on how to organize work are taken. A higher form of participation is labeled delegation, which includes employees taking part in work groups where they, without involvement, control the decision-making on how to organize specific work. An example of this in eldercare could be forming a work group for quality improvements in patient care and providing resources to support the group's autonomy in developing and implementing relevant measures. The highest form of participation in the ladder is labeled citizen control, which includes real participation and influence over all implementation steps concerning, for example, a project, including decision-making and funding resources [15].

In this study, our focus on the involvement and degree of participation of employees in decision-making processes can be compared to forms of delegation according to Arnstein’s ladder, i.e. employees having the power to organize specific work [15]. A DL differs in this context from general participatory leadership approaches by not merely involving employees in decision-making processes, but more specifically giving employees decision authority over how to organize and perform work. DL may also be compared to leadership made up of co-production approaches, where health interventions, service improvements, and applied research are co-designed and co-produced with patients, the public, and other stakeholders [16]. Even if some conditions seem valid for both DL and co-production, for example, the importance of paying attention to decent power-sharing among involved actors, the concepts differ as co-production does not particularly require the involvement of employees [16]. In this study, we have instead chosen to focus on organizational approaches that in more distinct ways include employees' involvement in decision-making and extended responsibilities in work. An example of this, included in this study, is nursing homes working according to the principles of internal contracts. Internal contracts mean that the municipality, represented by politicians as top management, still has the overall responsibility and control and that the nursing homes must adhere to the public framework of policies and guidelines, even though they enjoy freedom regarding their own budget, personnel, and development [17]. Hereby, first-line managers and staff are free to decide and control how planning, execution, and follow-up are carried out in their workplace, which gives room for development of DL among employees.

### Realistic evaluation framework

The study was based on a realistic evaluation framework to understand the mechanisms enabling or hindering the development and outcomes of DL. Applications of realistic evaluation framework mean ideally to hypothesize how mechanisms in program or change efforts may operate and contribute to outcomes in different contexts [18]. Pawson and Tilley [18] highlight the ambition to study what they refer to as generative mechanisms, meaning the factors causing certain things to happen in the context of, for example, change efforts. By studying the relationship between conditions (of different kinds), the mechanisms that are activated, and different forms of outcomes, it is possible to engage in an informed discussion on what causes things to happen or not happen (see, for instance, [19]). A critical aspect of the generative mechanisms of DL included in this study is how different workplaces choose to approach DL. The concept of *mechanism* is here defined in a relatively simple way and line with Danermark et al. [20]. Mechanisms are “what make things happen,” meaning the conditions, contexts (and activities) that generate (and can explain) events, social behaviors, and changes (see also [18, 21, 22]).

### Critical contextual factors impacting development and outcomes of DL

Overall, the Swedish labor context must be considered when, as in this study, analyzing efforts to achieve DL in Swedish eldercare. Sweden has a long history of employee involvement and collaboration between those leading and those carrying out the work. Traditions of distributive power were early manifested in industrial relations based on information and communication between employers and employees and were often presented as an illustration of the Swedish model. Already in the 70 s working life, the distribution of influence was given to self-organized work groups in the car industry [23]. Just as on the shop floor, labor relations in Sweden have been characterized by a short power distance between managers and staff in international comparisons [24]. This means that leadership styles accepted in Sweden are often characterized by styles aiming for joint actions through communication, influence, and participation (e.g., [25]).

Like many other countries, however, the governance of Swedish eldercare has been reshaped in recent decades, including market-inspired forms of governing and organizing care to increase the efficiency and quality of eldercare services [26, 27]. This has also resulted in increased top-down reorganizations [28], which seem to have contributed to a deteriorating work environment for care staff [29–31], including an increased workload [32–34]. Overall, studies point to Swedish eldercare struggling with difficulty in staffing, skills deficiencies, and a fragmented organization [35], while staff in Swedish eldercare also exhibit high rates of sick leave [36]. As a reaction to the negative outcomes of major top-down governance in health care, including overall negative working conditions, it has been suggested to introduce more trust-based principles to lead and govern health care [1]. Organizational approaches and efforts to develop DL can be seen as response to the call for more trust-based organizational forms. It is important also to stress that the implications of DL are of interest to policymakers within eldercare throughout different national contexts as the development of DL focuses on generic processes of how individual employees within organizations can share responsibilities including more optimal ways of using a collective experience base for delivering quality in care [37]

### Implementation of DL

The chosen focus on implementations of DL in this study represents planned activities or culture of DL that are top-down initiated and established by the strategic management in the studied organizations. Critical mechanisms contributing to the outcomes of DL concern thus *how* the strategic management has chosen to implement DL. A Swedish qualitative study of DL within eldercare argues for example that various forms of DL should be well-integrated into the organization to avoid opposition and conflicts in the organization. The study highlights the importance of formalization to ensure sustainable organizational support regarding the distribution of decision-making processes [14]. Another qualitative study performed in a Norwegian municipality on DL for integrated care in eldercare DL points out that organizational strategies and formalization of collaboration are pre-conditions for more spontaneous forms of collaboration in DL [37]. The focus mechanism in this study was thus formalization processes (mechanism A). Formalization in work organizations has been defined as declarations of procedures, rules, roles, and operation of procedures linked to work organization, including decision-making processes [38]. We have In this study, in line with Gronn’s [8] view on concertive action through collaborative structures, defined the formalization of DL as a guiding statement in terms of how forms of DL are organized in the workplace. Inspired by Hall et al.’s [38] indicators for formalization, this includes the degree to which DL is clarified by role descriptions as well as manifested in formalized authority structures and organizational procedures. More specifically, the formalization of DL could mean that the organization has clearly described which leadership tasks are delegated to employees (for example responsibilities for scheduling or specific quality improvement work) and that the delegation of leadership tasks to employees is formalized in the organization or based on a sustainable organizational structure facilitating employees' influence and participating in decision-making.

New formalized ways of organizing work, especially when initiated from the top, may, however, be resisted by employees. The importance of participatory approaches for the success of workplace interventions can in this context be pointed out [39, 40] and motivate our focus on *mechanism B (participatory approaches to implementation).* According to change management theory [41] common obstacle for development efforts is that employees have not been invited to participate in and contribute to the planning of re-organizations and thus do not understand why the re-organizations must take place or which objective is to be fulfilled. Kotter [41] claims that mentally, managers often exist “in the future and the vision” when communicating regarding development efforts, while employees reflect upon and communicate based on experiences in the present or, rather, the past. The importance of leadership enabling shared goals and visions has thus especially been stressed for developing more participatory approaches to change management [41]. However, participatory approaches to shared goals and visions also require that organizations understand and support the motivation and needs of first-line managers and employees to set aside time for learning and development [14, 42].

### Sense-making processes contributing to DL

Relational processes have been highlighted as critical for developing conjoint agency including alignment of views and activities of DL in an organization. Overall, it has been emphasized that DL is a leadership that functions relationally and develops in ongoing workplace and organization interactions [43]. Furthermore, interactions are anchored in specific contextual circumstances that shape a common understanding of this mutual responsibility-taking [44]. Overall, this means an emphasis on how leadership practices are constructed and reshaped in the interaction between leaders, workers, and situations, rather than focusing on structure, function, and performance of individual roles [43, 45]. Cooperation, trust, distribution of responsibilities, and decision-making processes are likely to mutually reinforce each other positively. Lusiani and Langley [46] elaborate on socially constructed forms of leadership in terms of “enabling leadership”, which focuses on practices, people, and tools through which a kind of coherence toward common direction emerges across different levels and sectors in everyday work life. To achieve a good implementation of DL, Marles [47] emphasizes in line with this reasoning the importance of “sense-making” [11], which includes creating the meaning of DL through collaborative practices. This refers to communicative processes and decision-making utilizing the synergy effect resulting from collective agency occurring when, for example, skilled healthcare professionals work together (e.g., [48]). Luciani and Langley [46] highlight a similar perspective, but the authors instead use the concept of "strategic coherence" to describe the emergence of mutual and compatible meaning in an organization. They argue that "strategic coherence" is socially constructed via activities that involve "refueling", "shaping" and "intertwining" mutual, compatible meanings. Luciani and Langley [46] argue that strategic coherence is a step in the development of joint meaning-making that involves natural participation from the grassroots in the organization. In the present article, however, we use the term sensemaking, but it is worth noting the similarities between these perspectives.

We have in this study chosen to differentiate between what we name “vertical sense-making” (mechanism C) and “horizontal sense-making” (mechanism D) to pinpoint the different critical aspects of the development of conjoint agency. In a vertical sense-making process we include meaning and views of DL being aligned, as an ideal according to Thorpe et al. [49], across different organizational levels. Using empirical examples, Martin et al. [10] demonstrate a disconnect between managers and professional workers in healthcare, including a mismatch in views on DL related to differences in leadership logic. Mechanism C could be exemplified by upper management of eldercare and employees in a specific nursing home share the same visionary goals of why and how DL should be implemented.

We have furthermore chosen to separately focus on processes of horizontal sense-making to pinpoint that DL could be seen as a “collective activity,” which is developed and manifested through close cooperation and mutual understanding between first-line managers and employees in work teams [see for example [2]. Previous research on DL in eldercare has also highlighted the importance of strong interpersonal relationships [37] and close cooperation between managers and their employees [3] as promoting factors for DL. Horizontal sense-making (mechanism D) can be exemplified by a team’s shared understanding of why and how to implement new ways of working according to the principles of DL, for example organizing work in self-governed teams.

## Methods

The study is part of a national research project on DL. The national research project has been approved by the Swedish Ethical Review Authority (Dir.: 2019–02934). Recorded informed consent was obtained from participants before all interviews and observations. Several articles and reports have been published within the project focusing on themes such as DL during Covid-19 [50] conditions for DL practices [2], development work in eldercare [51], and work procedural autonomy [52]. The publications address the issue of distributed leadership in different aspects and contribute Bidto knowledge on eldercare, trust-based management, organizations, and leadership. This study adds knowledge on mechanisms that enable or hinder the development of DL, and how the distribution of power, including participation and influence regarding decision-making processes and responsibilities increases trust. As a research team, we represent various disciplines within research including ergonomics, sociology, work science and social work. Additionally, the team possesses vast experience in conducting research and vast knowledge of eldercare. All authors hold a PhD; during the study, all researchers’ main occupations were senior lecturers at each university.

### Case study design

A multiple qualitative case study design was chosen for this study [53], which meant that six different organizational approaches for developing DL in eldercare were analyzed. This study is based on a case study performed during 2020–2022 in the form of interviews and observations at six specific workplaces in the field of eldercare in Sweden. All cases strived in different ways to work toward an organization emphasizing trust and distribution of power, including participation and influence regarding decision-making processes and responsibilities. The recruitment of workplaces was based on established contacts and known networks from previous research projects. From these contacts and networks, six different workplaces in eldercare were selected as cases as they, in different ways from a management perspective, seek to work toward an organization that emphasizes trust and the distribution of power and influence regarding decision-making processes and responsibilities. This means that the selected cases have in one way or another, top-down initiated, implemented, or are working with a more or less well-thought-out model for a sustainable and attractive work environment that includes some form of DL. The selection criteria may thus be described in terms of typical cases that are comparable. The cases are situated in different geographical areas in Sweden, representing both more and less densely populated urban areas, both private and public employers, and involve different occupational categories corresponding to different levels of formal education.

During the recruitment phase, the researcher shared relevant information for the study, such as reasons for doing the study, and previous personal experience of similar research projects. The participants were approached differently in the cases. In case 3, the researcher was doing participatory research and had already been introduced to the participants in an ongoing collaboration. In the other cases, the managers were approached by e-mail, and information about the study was given in the following individual web meetings with the researchers and managers before the data collection for this study. The researchers planned together with the managers how to contact a variety of employees with different backgrounds and different attitudes toward DL. In the second step, the researchers visited the workplaces and during these visits, face-to-face meetings with employees were held. Potential study participants were individually approached by the researchers and oral and written information about the study was given to all study participants before them consenting to participate.

The interviews were primarily conducted with first-line managers and with healthcare professionals carrying out the practical work. All authors have performed interviews and observations with divided responsibilities for performing the data collection in the different cases. During the conduct of the study, there were no dropouts and since participation was voluntary, only those who wanted to join the study participated. Several of the included cases have been studied previously in different contexts, which means that there has been a pre-understanding among the researchers performing the study. This applies in particular to case 1, which has previously been described during its project phase [14], case 3 [50] and case 4 [54]. The interviews carried out specifically in this study may thus in some cases be seen as follow-up studies focusing on the questions and the ambition underlying the analysis in this article. However, the workplaces are in different stages of development and their contextual conditions differ, which affects the implementation and nature of DL.

In case 3, the data were collected during the pandemic. Therefore, the interviews and observations were conducted digitally. The data in cases 4–6 was also collected partly during the pandemic and interviews with managers were performed digitally, while all interviews with employees were performed at their workplaces. All interviews in cases 1 and 2 were performed at the workplaces. Only those who participated in the study were present during interviews and observations. All interviews were semi-structured, allowing the researchers to identify key processes in achieving DL and at the same time allowing flexibility for the different cases to elaborate on contextual factors and outcomes significant in each case. The duration of the interviews varied between 35 and 90 min.

The interviews were semi-structured and covered topics on program theory, distributed leadership, and degree of formalization. Specifically, the following themes were in focus:

- What are the features of the program theory (i.e. how and why DL was developed)?
- Which measures have been implemented? How are these justified?
- Which effects are expected from the current measures?
- How do front-line managers experience the changes that have been implemented with a focus on trust, job satisfaction, and health?
- How do the care workers who work in eldercare experience the current changes based on trust, degree of co-determination, job satisfaction, and health?
- Which conflicts have arisen and why?

The above topics were covered in all interviews, but different follow-up questions were asked in each interview depending on the answers and getting an in-depth understanding of the mechanisms contributing to the outcomes.

All interviews were recorded and transcribed verbatim. In case 3, the transcripts were returned to the participants for comments or corrections. In other cases, the transcripts were not returned. Repeated interviews were not carried out in any of the six cases.

Observations were also performed of the cases, including attending different kinds of meetings where decisions were made. Physical and digital visits to the workplaces were carried out, including observations of work meetings, guided walks on the premises, and informal conversations with managers and employees. Field notes of visits and observations were made focusing on the same theme as in the interviews: program theory in practice, degree of codetermination, participation and influence in practice, and conflicts in practice.

### The analysis

The analytic process was characterized by an abductive process involving continuous interaction between theoretical concepts and empirical findings (see e.g. [55]). This meant that the empirical material (interviews and observations) was processed and structured in close alignment with various potential explanatory theories. Initially, the theoretical framework centered around Gronn’s [8] definitions of DL including concertive action and conjoint agency of DL. Based on Gronn’s definition of DL and the principles of program theory [56, 57] the step of the analysis focused on describing key stakeholders' ideal views and overall approaches to different forms of DL. This first step involved analyzing critical contextual factors for implementing DL, including degrees of formalization of DL and outcomes of DL such as employees' experiences of influence and participation in decision-making. A categorization of high or low degree of formalization of and involvement in DL was made in this step. The cases were categorized as having a high degree of formalization when there were coherent descriptions of structured ways of implementing DL including descriptions of formal procedures, meetings, and roles that clearly could be linked to DL practices [38]. Vice versa, a low degree of formalization was categorized when actors within the case did not exemplify forms and structures for sustaining DL. The high degree of involvement was categorized when it was described that a majority of staff were participating in practices that could be related to DL and the low degree when a minority of employees were described to be involved. Although not the primary focused outcomes, experiences of job satisfaction, well-being, and health were also considered when analyzing the overall outcomes and sustainability of the described DL.

In the second step, a realistic evaluation was used as an analytical framework [19, 20] of the mechanisms to identify and critically analyze mechanisms that enable or hinder the development of different approaches (i.e. program theories) to DL. This meant that efforts, and ambitions at workplaces to achieve DL were equated with different program theories regarding DL. In general, a program theory concerns the issue of how and why an intervention affects the participants of the program [56, 57]. This meant that we analyzed how the studied workplaces approached the realization of DL, how contextual factors interplayed with the approaches, and how this contributed to employees experiencing influence and participation in decision-making. This meant more specifically that the interplay between key processes for implementing DL and critical contextual conditions in the different workplaces was focused on understanding “what works?”; “for whom?”; “in which ways?”; “to what extent?”; and “in which contexts?” [18]. The analysis of the various cases focused on four central mechanisms (A-D) that influenced the outcome of the various attempts to implement DL, i.e. that made things happen in the various cases presented. The mechanisms A-D were theoretically identified (see motivations and definitions of mechanism A-D in the background) and explored in each case. Mechanism A, i.e. formalization processes, was in this study expected to contribute to the sustainability of DL over time. Mechanism B was expected to contribute a better adaptation of how DL is implemented to the needs and concerns of employees, as well as to a common understanding of the vision and goals of DL. It was assumed that mechanism C, vertical sense-making, would generate greater alignment across organizational levels on views on DL which would enable, and promote the development and sustainability of DL. Mechanism D, i.e. horizontal sense-making was assumed to be the key for implementing and sustaining DL within a work team. For each case it was analyzed if and how different processes and approaches that could be related to the mechanism were present in each case. Furthermore, for each case, critical contextual factors promoting or hindering the intended outcomes of different approaches to DL were considered. This meant in summary that it was explored if and how the theoretical identified mechanisms contributed to DL for each case. Finally, a comparative analysis of differences and similarities in the cases’ program theories, critical contextual conditions, and mechanisms was performed.

The analytical technique used to understand and describe the mechanisms that are central to understanding DL can best be described as a form of retroduction. Denmark et. alt [20] define the concept in terms of a "thought operation" that involves reconstructing the basic conditions for something to be what it is, or to put it differently, to analyze and elicit the properties that must exist for this phenomenon (DL) to be possible. In our case, it is about how, in what form, and under what conditions DL has developed in the various cases that we investigated. Another aspect of retroduction as an analytical tool is the use of counterfactual thinking, which means that we hypothetically ask ourselves questions where we omit various aspects of what happened and ask ourselves if the phenomenon would then have changed or remained the same. Through counterfactual thinking, we have utilized previous research to establish an understanding of which aspects are central to the understanding of DL. Retroduction is thus about trying to understand what factors must be present for the phenomenon in question not to cease to exist or radically change, and counterfactual thinking is the technique we use when we investigate this.

The descriptive analysis of approaches and outcomes of DL is first presented case by case in the findings. Secondly, the analysis of the mechanisms is presented one by one. In this part, diverse findings in all the different cases, are presented and elaborated for each analyzed mechanism. Overall, there is consistency between the data presented and the findings, and quotations are presented to illustrate the results. No analysis software programs were used in the analysis.

Instead, to manage a large volume of qualitative data on several cases, each case had a study protocol [53] based on the framework of realistic evaluation [18, 19]. Finally, the participants have not part of the analysis and have not provided feedback on the findings.

All authors participated in the coding and analysis process of the data with the main responsibility to contribute to the analysis of the data they had collected (see Table 1). Comprehensive insights into DL in each case were ensured by interviewing all first-line managers within the cases as well as inquiring interviewing employees in each case representing a variety of experiences and perceptions of the management principles applied at their workplace. Data saturations were discussed with the research team and finally decided by the researcher/researchers in each case when a comprehensive, nuanced, and coherent description of the DL in each case was achieved.

**Table 1:** Data collection in the six cases of the study

Cases	Participants	Interviews	Observations	Total data collected
Case 1 Researcher: GG	Managers Assistant nurses	1 manager 7 assistant nurses	Observations performed in a previous study^a^	8 interviews
Case 2 Researcher: GG	Managers Assistant nurses	2 managers 1 assistant nurse		3 interviews
Case 3 Researcher: ME	Needs assessors Unit managers	5 needs assessors 2 unit managers	25^b^	7 interviews and 25 observations
Case 4 Researchers: AE & MAB	Managers Team leader Team members	1 sectoral manager^a^ 3 unit managers 4 assistant nurses	3 full days of observations on the premises, including two steering group meetings, informal chats with employees and managers, and meeting residents of the nursing home	8 interviews and 3 days of observations
Case 5 Researchers: AE & MAB	Managers Team leader Team members Assistant nurses	1 sectoral manager^a^ 1 unit manager 5 assistant nurses 1 cleaner	1 full day of observations on the premises, including one steering group meeting, informal chats with employees and managers, and meeting residents of the nursing home	8 interviews and 1 day of observations
Case 6 Researchers: AE & MAB	Managers Team leader Team members Assistant nurses	1 sectoral manager^c^ 2 unit managers 4 assistant nurses	1 full day of observation on the premises, including observing one workgroup meeting and informal chats with employees and managers	7 interviews and 1 day of observations

^a^ The analysis of case 1 is also based on conclusions from a previous project [43], which relied on 82 interviews and continuous observations

^b^ In case 3, the researcher observed different kinds of digital meetings – unit meetings, workplace meetings, and eldercare meetings. The digital observations in the study did not include videos

^c^ The sectoral manager was the same person for cases Case 4, 5, and 6, and this interview was performed on one occasion covering aspects related to all three cases

## Results

In total, 41 interviews with managers and employees from all cases were conducted (see Table 1).

First, in this section, we present each case’s overall approach to and outcomes of efforts developing DL. Cases 4–6 are presented in one section as they are in the same municipality and share the same overall program idea. After that, the different mechanisms are discussed in more detail by comparing differences and similarities in the cases’ key processes regarding achieving DL. The overall results including the summary of the outcomes of each case and if and how each mechanism is manifested in the different cases are presented in Table 2.

**Table 2:** Categorizing main mechanisms and summary of critical contextual conditions and main outcomes of cases 1–6

	**Case 1**	**Case 2**	**Case 3**	**Case 4**	**Case 5**	**Case 6**
*Mechanism A:* Formalization	High degree	Low degree	Low degree	High degree	High degree	High degree
*Mechanism B:* Participatory implementation approaches	Dialogue and learning-oriented	Value and idea-oriented	Dialogue and learning-oriented	Value and idea-oriented	Dialogue and learning-oriented	Non-participatory
*Mechanism C:* Vertical sense-making	Some degree	High degree	Low degree	High degree	High degree	Low degree
*Mechanism D:* Horizontal sense-making	High degree	High degree	High degree	High degree	High degree	Low degree
**Outcomes**	Establishment of a trust-based organization with employees having a high degree of influence over their work	A work organization and culture characterized by self-determination and responsibility-taking among employees	Collective and user-focused work practices in work groups with a high degree of employee involvement in decision-making processes. However, a struggle to maintain time and structures for learning processes aimed at DL	Self-organized groups where employees largely are involved in decision-making processes including taking on leadership assignments. However, a struggle to maintain employees’ motivation to continue to take on responsibilities	Well-functioning self-organized groups where employees largely are involved in decision-making processes including taking on leadership assignments	An established structure of self-organized groups for DL but only a limited number of employees being involved in the work of the groups

### Overall description of the cases’ approaches to and outcomes of DL

#### Case 1

Case 1 was a residential care home located in a municipality in a larger Swedish city that participated in a major project aiming to create a new leadership practice, including new ways of organizing work in the direction of a trust-based organization and distribution of decision-making processes. Case 1 was in many ways ahead in the process toward more distributed management and decision-making. Already in 2016, a project was launched with the stated ambition to change leadership and organization in the direction of shared and joint responsibility. When the project ended in 2019, a completely new leadership training was created, and the staff had been further trained and prepared to participate in decision-making processes and governance. The basic idea of the project was that a changed leadership (in the direction of a coaching leadership) and increased influence for the staff would reduce sick leave, and staff turnover, and increase job satisfaction, which would subsequently make the employer more attractive and better meet the needs of recruits. The target group for the change that the project entailed was mid-level managers and assistant nurses. The key to this change was the new leadership training designed within the framework of the project as well as training for the personnel. The outcome of case 1 was a work organization consisting of several organizational structures based on trust principles aiming to establish a trust-based organization. These principles manifested themselves in expanded management teams and structured areas of responsibility for the staff. One of the unit managers described them as follows:

… then you have the management group with assistant nurses, which is made up of coordinators, and coordinators for different thematic areas. In each thematic area, there may be seven or eight representatives and they work independently in the thematic area with different tasks. And the assignments are given to them at the development meetings when an agreement is reached. (Unit manager case 1)

The involvement of employees in case 1 may be described as high, as the successful implementation of program ideas resulted in an established organization of trust with delegation of decisions and where employees, through formalized ways of organizing DL, participated in decision-making processes. After the project was completed, lower sickness absence rates and reduced staff turnover could be observed. The consequence of the changes and the distribution of responsibility carried out in case 1 was reported to have led to increased self-esteem and commitment to the work and also the experiences of the workplace being more attractive. One of the employed assistant nurses described it as follows:

… so you get professional status and job satisfaction … you feel that it's not just someone who sits and points with their whole hand … you feel seen … (Assistant nurse, case 1)

#### Case 2

Case 2 was a residential care home organized by a nationwide non-profit Christian foundation. The principles of the foundation’s work model were largely in sync with the vision of DL characterizing the work at residential care homes, as it is largely based on decentralized self-determination and focuses on older persons. The expressed goal was to introduce a responsibility-based organization emphasizing the mission and the residents (guests, care recipients, etc.). The overall goal was described as realizing and developing a change in mentality among the staff, a mentality that has a user focus and where responsibility for decisions and measures is decided through conversations and developing ideas. One of the managers expressed it as follows:

…fundamental values are summed up with “the will to see and the power to change”. And those words say a lot, I think. They set the foundation and basis for how we work and how we should think. (Operations manager, case 2)

This idea of a responsibility-based organization was described as rooted in the entire organization, but there were no specified activities or structures for how to reach these goals of DL. In other words, were decisions made as close as possible to those who perform work? Employees also experienced close relationships with management and managers. Staff turnover was very low and working in the organization was perceived attractive, largely due to the clear vision where users are in focus. The involvement of employees in decision-making processes may thus be described as high and value-based with a great focus on the users. Formal activities to achieve DL were not pronounced but were the management rather aimed at developing a culture of responsibility-taking and self-determination among employees. Employees also experienced close relationships and communication with managers and management which facilitated for conjoint agency of DL within the case.

#### Case 3

Case 3 was a social service unit responsible for assessing needs in eldercare located in a municipality in the capital of Sweden. Case 3’s approach meant that DL was not manifested in a specific form of organization but rather involved collective activities and practices, mainly constituted as ongoing learning regarding the needs of older people in the community and changes in law, practice, and guidelines. Influence and decision-making were found in ongoing interactions between managers and needs assessors. The DL was more specifically executed in teams where one needs assessor is a group leader, and the groups are self-organized in terms of meeting structure and sometimes how the development of knowledge was to be conducted. The group later distributed knowledge to the rest of the needs assessors in the unit. The group work aimed at specific learning and further training regarding different needs or vulnerabilities among older people in the community. This form of user orientation was based on the professional role and norms strongly linked to ideas of advocacy and user rights.

Case 3 can be categorized as exhibiting a high degree of involvement of employees in DL. DL practices were a vital factor in the ongoing work with developing the organization’s leadership work and were shaped through collegial discussions and group work based on the needs assessors’ knowledge of the target group. One needs assessor described the ongoing discussions in the following way:

And find out things together, as it usually happens. Today I might consult a colleague who has worked longer than me. Or maybe I ask the question so that everyone can hear. It's easier if you've been through it, then you can solve it when it happens. (Needs assessor, case 3)

There was a high degree of DL, constituted by learning processes and needs assessors overall experienced an ability to influence and participate in leadership work. However, problems with the meeting structure, outdated and inadequate administrative guidelines/regulations, and weak information channels hinder the possibility of achieving DL.

#### Case 4, case 5, and case 6

Cases 4, 5, and 6 shared the same program ideas on DL as they represented three nursing homes situated in the same medium-sized municipality, sharing the same principles of how to organize work (i.e., the principles of internal contracts). The organizational form of internal contracts meant that first-line managers and staff in the cases enjoy freedom in terms of budget, control of planning, execution, and follow-up of how work is carried out in their workplace. At the same time, they also had more responsibility for the consequences of their collective management. For example, if there was a surplus in a nursing home’s account at the end of the year, the unit could keep the money and spend it at its discretion, as long as it is done legally. On the other hand, if a nursing home did not successfully balance its budget, the unit needed to take action in line with a deficit. The motivation for implementing internal contracts sprang from the idea that a high quality of living is related to older residents influencing their lives to pursue habits of their choice with help from staff and that the staff, in turn, have extensive room for decision-making and responsibilities to facilitate a high quality of living.

All three cases were governed by principles of distributing leadership through self-organized groups with specified areas of responsibility and through a formal management structure where traditional management tasks were delegated to employees. This meant that upper management had decided on a fixed structure for how to organize for DL. The self-organized groups had a mandate to decide on planning and organizing work, including influence over the budget and how to use available resources in ways that benefit the common good for both residents and employees. A fundamental idea was that all staff should choose and participate in one of the self-organized groups linked to specified tasks and responsibilities, including arranging activities for residents, scheduling staff, care quality procedures as well as purchases and budget work.

Another assistant nurse in case 5 described how she, after the implementation of the internal contract, was able to get insight into and control budget spending and that the increased control also was a reason for why they today were in a surplus:

…but you have insight and see where the money [goes]… before they [the money] just used to disappear. We got to hear all the years [before the internal contract was implemented] from the municipality that we had a budget deficit. […] but during the recent purchases [since internal contracts were implemented] the residents have participated in the decisions on what we should buy, crystal chandeliers and outdoor furniture…. (Assistant nurse, case 5).

Representatives for each working group were appointed and they, in turn, formed the management group of a nursing home. The representatives were leaders with no formal mandate but with skills/capacity to guide, coach, and push colleagues to make them learn and assume tasks for the group. Furthermore, they acted as spokespersons while describing intentions, initiating decision-making as well as listening to attitudes and suggestions from colleagues, which they put forward for the management group to handle.

The pioneer nursing home, case 4, initiated the principle of internal contact in 2010, more than a decade ago, while the other two, cases 5 and 6, followed with four years between them.

The actual employee involvement in a DL differed between the three cases depending on the extent to which the different cases had managed to secure independent active work and participation in the self-organized groups. The program idea was successfully implemented and was well-regarded among employees, expressed in the following way by one of the unit managers who started as an assistant nurse in the municipality:

The influence also makes you want to stay, so… [This is] the first time I have stayed in a municipality. I usually continue after two, or three years, but I did not do it this time [laughter] when you got the chance to be involved and influence (Unit manager, case 4).

There were, however, some challenges and tensions in case 4 in recruiting new members to the self-organized groups, including leadership tasks seen as more burdensome, which can be seen as a threat to the sustainability of the intended forms of DL. The involvement in case 4 may thus be described as high but struggling with employee involvement.

In case 5, employees expressed in interviews a high degree of joy and satisfaction related to their work, the management of the nursing home, and their participation in the self-organized groups. The involvement of employees in case 5 in terms of DL may be described as high with a high degree of employees participating in the intended and formalized ways of organizing DL through self-organized groups based on program ideas. There was also a high degree of expressed engagement and desire among employees to participate in decision-making processes.

In case 6, on the other hand, most staff were resistant concerning the principle of internal contracts. At the time of the study, management struggled with recruiting employees to the self-organized groups, resulting in a low level of involvement among employees when it came to realizing the idea of delegating management tasks. One of the few assistant nurses being engaged and taking on leadership assignments described the low engagement in the following way:

There have been a lot of difficulties along the way, there are many employees who not really are on board, they are somehow in their little words… and are not able to change their minds (Assistant nurse, case 6).

The involvement of employees in case 6 may, in summary, be described as low, as the organization formalized ways of how to implement DL through self-organized groups, while employees were only to a limited extent involved in decision-making processes in these groups.

### Summary of outcomes

**Fig. 1:**
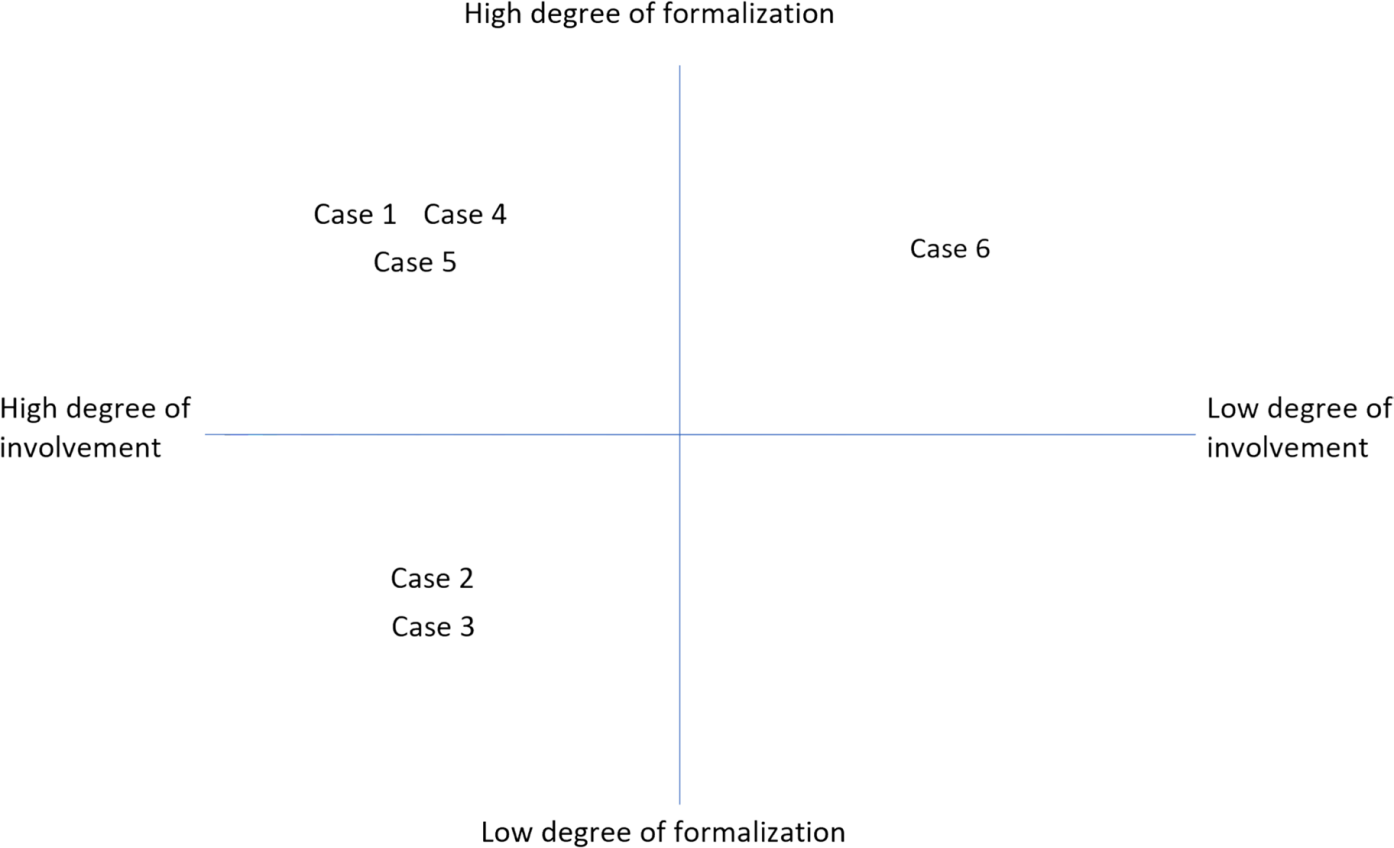
Categorizing cases according to the degree of formalization when realizing distributed leadership and the current degree of employee involvement

The six cases examined in this study had all been embedded in different contextual conditions and were found at different stages in the change process seeking a more distributed leadership and a more trust-oriented organization of work. To summarize, five of the six cases may be described as having distributed leadership to employees. In these cases, outcomes of distributed leadership include experiencing a high degree of employee involvement (see Fig. 1).

In cases 1, 4, and 5 participations in decision-making processes and responsibility-taking were experiences through formal structures of organizing work. In cases 2 and 3 self-determination and influence over decisions were rather experienced through shared organizational values expressed through an organizational culture or professional norms that influenced participatory and collaborative ways of working. Some challenges in sustaining the achieved DL could however be identified in cases 3 and 4, which is more in detail elaborated on below when outlining the mechanisms contributing to DL.

### Mechanisms contributing to the outcomes

Formalization and participation in the change process were two important aspects of implementation work, but we can also state that horizontal and vertical sensemaking greatly influenced the outcome. Table 3 summarizes enabling factors and key barriers for each of the four mechanisms including representative quotes from study participants illustrating the mechanisms and key factors. We will go below through these four mechanisms in more detail before taking the step over to a summary discussion and conclusion.

**Table 3:** Summary of and representative quotes related to enabling factors and key barriers for each of the four mechanisms

Mechanisms	Enabling factors contributing to DL	Key barriers	The importance of the mechanisms across the cases
***Formalization processes*** Representative quote: *For me, trust organization means that you set up supporting structures to be able to delegate and to be able to create real influence and participation and not just with words … and trust-based leadership … yes … I like the word distributed leadership better. Trust me… you can't just think it's a “let go” or drop the ball … and see where it lands … I think that's a way to abuse people … So… for me, it's much more about supporting my employees to succeed in their assignments so that they can feel proud.* (Unit manager, case 1)	Providing structures and processes manifesting how to realize the principles of DL Being supporting structures and processes for sustaining DL over time Representative quote: *We have our shares and our responsibilities […]. One is responsible for booking music. I am responsible for the activity brochure. [Name] and I are responsible for the garden. So each us of have or things so to say*. (Assistant nurse, case 5)	High degrees of staff turnover, sick leave, and high workload Limited interest or resistance among employees to participate in meetings and processes including non-traditional work assignments Representative quote: *It is not ok [that we spend spare time on work] but we do it because we want to make it work. I have spent a lot of my spare time for the years on project work. I have always waited that it…one day just will be a good flow, but it does not seem like that [will happen].* (Assistant nurse, case 4)	Referred to as important aspects of DL among most interviewed persons in cases 1, 4, 5, and 6 More limited elaborated on in cases 2 and 3 which also had low degree of formalized processes of DL. In these cases, the culture of DL and professional norms related to DL were rather stressed
**Participatory approaches to implementation** Representative quote: *.. you feel noticed and you feel that what you do … well, you kind of get feedback … everyone has quite a lot of freedom because the managers don't get involved. And I feel that I've been able to grow with my role, that I've been able to find out information and educate myself and sort of… well, without any pressure sort of and grow into it… …so you get a professional status and job satisfaction when you.* (Assistant nurse Case 1.)	Stepwise engagement of employees Implementation through dialogue and learning-oriented approaches Managers coaching approaches Value and idea orientation giving a common aim on how and why to implement DL Representative quote: *Yes, then she [the unit manager] was very active and she sat down with us in the groups and informed us what we should do, and… well, there was a lot of help you needed, a lot of information to be digested. So, she supported us the whole time, all the way.* (Assistant nurse, case 5)	Negative attitudes among employees to implement new and distributed ways of organizing work Contextual factors (e.g. budget deficit) contributing to resistance to taking extended responsibilities in work Representative quote: *They still express it like” Yes, but we did not choose this”. This is something we can’t bypass, and I also think that is the reason we did not get it kicking in the same way as [case 4 and case 5]* (Unit manager, case 6)	Aspects of participatory approaches as a critical mechanism were expressed in all cases and elaborated on in most interviews
***Vertical sense-making*** Representative quote: *It started with politics… They [the politicians] were so interested in Thyra Frank [a leader who organized a care home similar to the principles of internal contracts] in Denmark. …they saw [when doing study visits in Denmark] a great, great warmth [between employees and residents in the care homes]. Yes, and that you hugged each other. […]…empowerment … that comes from within. The politicians gave them a free arena to plan their business as they wanted. Allowed the assistant nurses to a large extent to participate and influence. [Gave influence over] “the how”. And this was a big driving force for the assistant nurse group, you felt that "Yes, but this is possible to do. How can we achieve this?” And then [at case 4] there were some driven assistant nurses who felt that "We have to start doing this". …how would we have the groups so that it would be a feeling of home, cordial and nice". Many assistant nurses have shined in their roles when they receive the assignment* (Sector manager for cases 4, 5, and 6)	Clear change idea that is supported at different organizational level Strong support from top management Common values within the organization that align with DL Representative quote: *…once you have the context, it is a fantastic employer, precisely because it is not for profit, and you have a strong belief in team spirit. It's not about the boss being able to do better than someone who has another so-called lower position, instead, we learn, and we see each other all the time regardless of position. It is a team feeling.* (Unit manager, case 2)	Upper management not listening to employees’ concerns including poor work environment conditions A lack of trust and collaboration between organizational levels Representative quote: *… the first line management and key actors higher up in the organization did not agree [with each other how the change should be implemented] so … there was not clear support [from the top of the organization]* (Unit manager, case 1)	Dimensions of vertical sense-making were explicitly mentioned in cases 1, 2, 4, 5, and 6 All managers in the different cases lifted aspects of vertical sense-making. Related aspects were also expressed among some employees, especially when there was a lack of vertical sense-making
***Horizontal sense-making*** Representative quote: *We've kind of worked well together and… uh, we've helped each other and…, supported each other. So that… when someone new comes, you kind of get to help and train them in the different systems that we use, different rules and so on, so that they join the gang. So I still think it has worked, it has been a good group. […]. We kind of had fun, if you can say that [laughter]* (Assistant nurse, case 5)	Strong social cohesion and a team culture of trust and collaboration Regular team meetings including collaborative and learning processes aiming at DL Representative quote: *….if you have a good community in your work group, most things work. And we have that here. […] you are considerate of each other, and you listen to each other and give each other respect.* (Assistant nurse, case 5)	Employees prefer a more hierarchical work organization Lack of vertical sense-making hindering teams’ progress in developing DL Re-organizations hindering the continuation of group and learning processes Representative quote: *But then they [my colleagues] think that [doing quality work] is the job of the manager, that it is not my job [as an assistant nurse] to do.* (Assistant nurse, case 6)	Aspects of horizontal sense-making were elaborated on in most interviews with employees and in some interviews with managers In cases 2, 4, and 5 strong social cohesion and team culture of collaboration were to a great extent stressed The collaborative learning processes within teams facilitating DL were mainly expressed in case 3 In case 6 horizontal sense-making on DL was not established as employees preferred a more hierarchical organization of work

### Mechanism A: formalization processes

All cases had a clear and well-thought-out idea with more or less formal structures supporting the implementation. Four of the cases (cases 1, 4, 5, and 6 see Fig. 1) may in this context be described as having program theories characterized by a high degree of formalization of DL, including management structures and a formal organization for DL. The cases draw on, for example, specifically designed leadership training with a focus on distributed leadership, formal organizing of efficient group work, and other structured elements enforcing trust-based organization. The results highlight that formalizing DL created a structure for sustaining DL over time and thus a mechanism contributing to the outcomes in cases 1, 4, and 5. In case 1, where they had worked with a conscious formalization of various distributed functions one of the unit managers described it as in the quote presented in Table 3.

In contrast, contextual conditions in case 6 indicated that the structures and plans for formally organizing DL were not sufficient for achieving DL. More specifically, the results showed that strict forms of formalization may constitute a hindering factor if there are no organizational champions or if the organizational form does not match employees’ maturity or experienced situation. In case 6, there was strong employee resistance regarding participating in fixed forms of self-organized groups shouldering distributed responsibilities. The case lacked staff willing to take on responsibilities beyond traditional work tasks, which interacted with a strong formalization of DL. The same tendencies could be noted in case 4, where there were well-functioning forms of institutionalized DL overall, but where there was a struggle to motivate new employees to take on leadership responsibilities. Even though the case was characterized by a DL this nursing home had lately experienced a backlash in terms of challenges to recruit new employees to the self-organized groups, especially for responsibilities perceived as “heavier,” such as quality and budget issues. One of the engaged and experienced assistant nurses described also an overall context of being understaffed and that the DL also became a burden when a more limited number of people were engaged (see the related quote in Table 3).

Cases 2 and 3, on the other hand, developed a DL without any form of formalization. The mechanisms contributing to sustaining DL in these cases may rather be related to the internalized organizational value favoring principles for DL (case 2) and employees’ strong client engagement (cases 2 and 3). The specific contextual conditions in case 2 included a value-driven workplace, operated by a value-based foundation. In this contextual environment, it was possible to select employees willing to take on leadership responsibilities without institutionalized forms. Management in case 2 benefited from the organization’s good reputation, which facilitated recruiting suitable personnel. The DL in case 3 was dependent on the engagement of individual employees, rather than supported by formal structures facilitating the aim of a more distributed decision-making process. This engagement was linked to the professional role and ideas on advocacy and user rights rather than internalized organizational core values (cf. case 2). One need assessor described the driving forces of DL in the following way:

But this is also the case when we work with people. Because no cases are the same. That's where it becomes important with our collegial case reviews because then we help each other to find a solution in each case. And then there are always new case law and so on. So, the right practice yesterday is not necessarily the right practice today. Because as soon as a new precedent-setting judgment comes out… And that's why I think you're in an ongoing process all the time. And the managers can't have all the answers, because they don't have all the information all the time. (Needs assessor, case 3.).

The function of the first-line managers in case 3 was mainly to facilitate the needs assessment through meetings and meeting structures, information, and guidelines as well as making room for discussions, further training, group work, and collegial knowledge exchanges. This aimed to distribute decision-making in needs assessments to the needs assessors possessing the required knowledge regarding the needs and situation of older people in the community. In the organization as a whole, however, there were contradictory ideas that, along with poor working conditions and high staff turnover, weakened any formalization of DL. All in all, these contextual factors in case 3 hindered the continuation and creation of structures for sustainable learning processes aimed at achieving DL.

### Mechanism B: participatory approaches to implementation

Mechanisms contributing to DL may also be related to the degree of participatory approach regarding *how* DL was introduced and implemented in interaction with critical contextual contexts. The identified participatory approaches included *dialogue and learning-oriented approaches* through which cooperation, participation in organizational changes, and shared responsibilities enabled the distribution of leadership throughout the organization. A participatory approach was furthermore facilitated by a *value- and idea orientation* giving a common aim on how and why to implement DL.

Cases 1, 4, 5, and 6 all to some degree had a top-down approach as upper management had decided on a more or less fixed structure for how to organize for a distributed leadership. Aspects of both dialogue-orientated and learning-oriented approaches could be seen in the implementation process of 1, 4, and 5. In case 1 the participatory approach was, for example, expressed in the leadership training that was launched and in a completely new managerial role characterized by dialogue through coaching, support, and delegating responsibilities, as well as through the introduction of extended management groups that also included staff. Another aspect underpinning the dialogue-oriented participatory approach was that the employees continuously utilized reflective teams that included management, assistant nurses, and other employees.

The slow but also well-structured and decisive and dialogue-oriented process toward a more distributed leadership meant that the staff was both positive and well-prepared for a development program aiming for increased shared responsibility in terms of how the work was organized.

In both cases 4 and 5 employees were initially skeptical about implementing internal contracts in their nursing home. However, an important mechanism contributing to the gradual engagement of employees concerned how the program ideas were implemented in dialogue through learning-oriented approaches. Additionally in the pioneer case 4 the implementation process had a strong value orientation with a strong focus on creating good living conditions for the residents which increased employees' engagement and willingness to participate. In case 5 employees were initially more skeptical, but the case started with a trial period and the employees could afterward choose whether or not to keep working according to the principles of internal contracts. It was described that during the trial period, the working principles were more thoroughly described and justified by managers and heads of teams, which contributed to convincing most employees to want to work according to the principles. In case 5, a critical implementation mechanism involved the manager taking an active role in coaching and facilitating employees engaging in self-organized groups. The manager’s approach to coaching and motivating employees to take on responsibilities positively interacted with the nursing home being a smaller unit in terms of the number of employees.

In case 6, on the other hand, several years after implementation, most staff were still resistant regarding the principle of internal contracts, as they felt forced. The nursing home even had some employees who previously had quit from other nursing homes to avoid working according to the principles of internal contracts. Unlike case 5, case 6 did not have a trial period where the employees themselves could decide whether to continue working according to internal contracts. The decision to implement internal contracts was simply a management decision without any participation of employees, leading to the latter feeling as if they did not have much of a choice when it came to the nursing home signing up to work according to internal contracts.

At the time of the study, management was struggling with recruiting employees to the self-organized groups, meaning that there was a low level of involvement among employees in terms of realizing the idea of delegating management tasks. In case 6, contrary to cases 4 and 5, the managers participated in the meetings of the self-organized groups, leading and structuring the work of the groups. A few employees in case 6 had committed to being responsible for management assignments (e.g., quality procedures), but this was described as an assignment being performed in a more solitary fashion and not in the self-organized groups. The few employees who described being engaged in the implementation of internal contracts expressed that their engagement was not popular among their colleagues.

In comparison, negative attitudes in case 6 may be related to contextual factors such as the nursing home exhibiting a more negative financial situation compared to other nursing homes in the municipality, thus making employees hesitant when it came to being responsible for budget decisions, not feeling as if they were or being able to decide on how to use a surplus, like other nursing homes. In case 6, a limited participatory approach for implementing DL with a strong resource approach contributed to employees being hesitant about being responsible for budget deficits. In cases 4 and 5, more of a coaching and participatory approach in a less strained financial and a more collegial collaborative context made the resource approach more attractive, as employees were able to enjoy making decisions on how to use the surplus.

Cases 2 and 3 did not embrace clear implementation strategies of DL, but the implementation of DL could still be interpreted in terms of participatory processes. The work in case 2 was largely value-driven with a strong focus on the residents based on the values of the non-profit organization owned by the foundation. The principles of DL were manifested in the organization’s basic ideas in terms of being a non-hierarchical organization with a generally high degree of participation and responsibility among employees. The organization may be defined as flat, and the degree of flexibility and individual adaptation was high. The fact that the organization is based on a Christian view and characterized by a very close and non-hierarchical relationship between management and staff meant that DL, including participatory decision-making processes, permeated the entire organization. The basic values including a focus on users and joint decision-making also made the workplace in question very attractive for staff, which is not least indicated by a very low level of staff turnover. This good reputation meant that the organization could employ the most dedicated healthcare professionals, thus facilitating the distribution of responsibility and decision-making.

Finally, the approach to implementing DL in case 3 may be largely summarized as a *learning orientation.* Their learning approach differed however from other cases by more narrowly focusing on further training about the needs of the target group. There was, for example no ongoing collegial dialogue on how the work is to be organized, nor were any collegial decisions made aiming to strengthen the DL itself (e.g., about decision-making, case management, or personnel matters). Additionally, strained resources due to budgetary cutbacks, high staff turnover, and high sick rates make it hard to obtain a dialogue orientation. Instead, DL developed organically in that needs assessors were the ones possessing knowledge of the target group thereby influencing the focus of the work using a participatory approach.

### Mechanism C: vertical sense-making

Another important mechanism contributing to outcomes could be summarized as the vertical sense-making processes that to a greater or lesser extent were taking place in cases 1, 2, 4, and 5. The vertical sense-making was in these cases developed through collaborative processes within and across different organizational levels, for example across work teams and higher organizational levels. A critical contextual condition for developing vertical sense-making in all mentioned cases was strong support from top management being active in initiating, promoting, and communicating the change idea on how to realize DL.

In case 2 the vertical sense-making was closely related to the decentralized culture outlined above under the description of mechanism B. The contextual factors characterizing the organizing of work were above all the close relationship between management and staff which facilitated the vertical sense-making of the understanding of the organizational values including employees´ self-determination and responsibility of working according to the values (see example of quote in Table 3).

Relationships across organizational levels and a high degree of personal involvement and decentralization were in case 2 prioritized over formalization and routines (compared with the content of mechanism A). These aspects seemed in the case favorable about the informal distribution of responsibilities and influence (i.e., that the staff share and internalize the values characterizing the organization).

Case 1 had a clear and well-thought-out change idea and dedicated project managers supported by senior managers including the city management. At an earlier stage, the project had received funding from the EU, which created good financial opportunities to develop and implement the training that was considered necessary for the implementation. These were all important conditions and vertical sense-making processes were initially facilitated by the fact that it was possible to create a project organization where managers and staff received training and together developed an arena for reflection and dialogue. This meant that a learning process was initiated at several different levels contributing to the preparedness, support, and legitimacy of the change ideas. However, after a while vertical sense-making was hindered, as collaborations and trust across organizational levels had not been developed simultaneously which meant that DL after being initiated was maintained as more restricted to a team level with no functioning collaborations between organizational levels. Over time the change efforts also met with some resistance in the form of conflicts between different levels of the organization, as mentioned by one of the unit managers (see Table 3 for quote).

In connection with implementing the project idea in case 1, it was difficult to form interest alliances with other central functions in the political administration. This created resistance and irritation that prevented the idea (concept) from spreading further in the organization. A gap arose between the HR function and the project management, who to some extent felt that they were not given access to the work of creating a trust-based workplace characterized by distributed leadership. However, these problems were eventually successfully overcome thanks to a clear idea of change and a high level of acceptance and commitment among the ones representing the target group of the change.

Like case 1, cases 4, 5, and 6 were implementing a clear and well-thought-out change idea that had strong support among politicians within the municipality, the sector manager of eldercare also being a champion for the implementation of internal contracts as well as unit managers and assistant nurses being interested in work according to the concept.

The managers of cases 4, 5, and 6 had the same sector manager and had a common platform for supporting each other and driving the change by all being part of the same management board of eldercare in the municipality. This created conditions for managers at different levels to be dedicated champions for the change idea and play active roles in vertical sense-making processes including promoting, explaining, and creating conditions for the change ideas at different organizational levels. Case 4 had over the years more specifically been highlighted as a schoolbook example where managers early on championed the concept of internal contracts with the vision to create a home-like environment with a high quality of care for the residents. Being lifted as a good example reinforced the common view of the value of working according to the principles and smooth collaboration with employees regarding business-related questions, including a good collaboration with union representatives were described. An important context was having committed and stable staff, having worked there for a longer period, who felt strongly about improving the lives of the residents, and thus employees over the years also became champions for the realizing of internal contracts. In case 5, where employees initially were more skeptical about the implementation of DL and some assistant nurses even quit when it was decided to work according to the principles. The presence of a charismatic, highly appreciated, and engaged unit manager played, however, a critical part in supporting vertical sensemaking processes through coaching employees to take on management responsibilities. However, even if case 6, being in the same municipality as cases 4 and 5 and sharing the same context with top-down support including managers being champions for internal contracts the vertical sense-making was more limited. Managers struggled to promote employees´ participation in the self-organized group but the resistance among employees limited vertical sense-making processes on the implementation of the change idea. The social processes contributing to the limited implementation are further outlined below under mechanism D, horizontal sense-making processes.

In case 3 mainly horizontal sense-making could be found, elaborated more on below under mechanism D. Bottom-up driven attempts of advocacy for change could be seen as the unit manager used the work groups’ concerns related to user orientation upward in the organization in her criticism regarding lacking resources and deficiencies in the organization. However, this did not contribute to vertical sense-making processes as the concerns to a more limited extent were addressed higher up in the organization. Overall vertical sense-making processes were also hindered by high rates of sick leave during the pandemic, staff turnover, and a heavy workload represented for the stability in forming collaborations within teams as well as between different organizational levels.

### Mechanisms D: horizontal sense-making

The results pointed finally at horizontal sense-making, including co-creating shared responsibilities in the work group, as a critical mechanism for developing and sustaining DL at the team level. In these cases, work was mostly performed in collaboration with team members, and the results point to the role of well-functioning decision-making processes in the work teams or the work teams’ support of the individual’s decision-making. With less involvement of formal management, the teams’ sense-making on how to make decisions became critical for how DL was developed in the different cases.

In this context overall a strong social cohesion and team culture of collaboration seemed to facilitate the sense-making of decision-making in work teams, which was especially exemplified in cases 2, 4, and 5. In case 2, the contextual factors characterizing the work were above all the close relationship between management and staff. This relationship and a high degree of personal involvement and decentralization were prioritized over formalization and routines. These aspects seemed favorable about the informal distribution of responsibilities and influence, i.e., that the staff in teams shared and internalized the values characterizing the work. In cases 4 and 5, DL was organized through self-organized groups, including management tasks. Responsibility and decisions were in these cases often made through consensus in the teams as no formal leaders were appointed. One part of the horizontal sense-making process included in this context is how to share and take on increased responsibilities among employees. In case 4 assistant nurses with long experience of being engaged in the self-organized groups described how their organization of work required employees to step forward and in a reciprocal process take on different and complementary areas of responsibilities. In both cases, 4 and 5 experienced nurses also described how they were fostering and teaching new members in the self-organized groups how to approach and execute new areas of responsibility. In case 4 overall positive collaboration between managers and employees was described, but also tensions between employees and managers and group dynamic challenges related to sustaining a well-functioning DL over time were mentioned. In case 5, employees more consistently described that they experienced the nursing home as characterized by collaboration, trust, and a good atmosphere between employees. In case 5 it was highlighted how consensus processes were facilitated by strong social cohesion and a positive social climate, both between team members and between the closest managers and team members. Employees also expressed just how satisfied they were with the psychosocial environment at the nursing home.

In contrast, case 6 did not manifest a strong collaborative culture between managers and employees. Managers described an immature work group who preferred a more hierarchical work organization in which managers, not employees, should be responsible for performing traditional management tasks, including scheduling, budget work, etc. In interviews with employees, they described a certain distance and even distrust of managers. One of only a few employees who were positive and took on increased management responsibilities described how other employees in a negative way referred to her as a “mini manager.” The strong resistance also made it hard to recruit members to self-organized groups. Thus, the work teams' sense-making processes seemed more concerned with a joint understanding of the negative aspects of the implementation of DL in the form of internal contracts.

In both cases 1 and 3 the horizontal sense-making processes promoted the development of DL within work teams but were also to some extent restricting the sustainability of DL, as described above in mechanism 6, when not being combined with vertical sense-making. In case 1 the strong dialogue-oriented approach that was manifested in the relationships between first-line managers and employees facilitated the horizontal sense-making on how DL should be realized within the work teams. However, as outlined above under mechanism C, collaborations and trust across organizational levels had not been developed simultaneously which meant that DL after being initiated was maintained as more restricted to a team level with no functioning collaborations between organizational levels. Resistances and conflicts across organizational levels had to be overcome to sustain a functioning DL over time, i.e. there had been a need for vertical development of a common vision that extended up to the top management to ensure stability and long-term sustainability. However, such a vision was partially lacking.

In case 3 the DL was constituted by group work and learning processes aimed at specific learning and further training regarding different needs or vulnerabilities among older people in the community. The learning processes strengthened collegial relationships and horizontal sense-making on how to make decisions with the user in focus including joint criticism of working conditions that make a user orientation difficult. The results in this case pointed to how having the support of the group and common sense-making processes in group meetings on how to approach individual decision-making, may make the individual employee less vulnerable when it comes to being the only one responsible for management tasks and decision authority. Case 3 also highlighted the importance of stability in terms of team members as well as the overall organization to achieve continuity in the collaborative and learning processes facilitating DL. In the needs assessor group, there were a few employees with long working experience in needs assessment. This was important to maintain continuity and to share work experiences on needs assessment in the community. The study was however conducted at a time of several organizational changes, including a fusion of two units and changes in terms of organizing first-line management, including changing managers. This challenged collaborative processes and to some extent hindered the development of DL in case 3. During the study, there were several new employees at the workplace, and a few employees chose to quit. Staff turnover led to a never-ending introduction of new employees and a tiring process of starting over which hindered both the horizontal sense-making processes and the sustainability of DL.

## Discussion

This study contributes knowledge on how workplaces in eldercare approach the realization of DL including how different mechanisms enabled or hindered the development of DL. Aypay and Akyüred [13] address the issue of this demand for studies of DL approaches in practice. This study has critically focused on DL, its forms, and mechanisms from the perspective of a realistic evaluation framework [18]. This means in terms of Gronn [8] that one important overall mechanism was a shift in focus from leaders to leadership activities and a shift to driving forces, i.e. in this study DL’s activities and driving forces that contribute to DL including performing work with a user-orientation.

By focusing on the mechanism of activities and process, it became apparent how roles, power, and definitions of reality in the cases were negotiated and constructed in specific social interactions and what characterized these interactions (cp. [58]). To focus on processes of DL is furthermore a matter of visualizing critical aspects that contribute to as well as hindering outcomes in the workgroups in question, and how these aspects interplay with actors, such as health care professionals, older persons, and formal managers, in different kinds of Swedish eldercare contexts. The study especially contributes knowledge on how the four mechanisms of *formalization processes, participatory approaches to implementation, vertical sense-making*, and *horizontal sense-making* contributed to the outcomes of efforts to implement DL effort. These are vital insights into how eldercare organizations can improve the quality of eldercare and user orientation.

The mechanism of *formalization processes* was possible to identify by different forms of DL encompassing workplaces with a high or low degree of formalization in terms of developing DL. Inspired by Hall et al.’s [38] indicators for formalization include the degree to which DL is clarified by role descriptions as well as manifested in formalized authority structures and organizational procedures. Four of the studied cases (cases 1, 4, 5, and 6) exhibited these kinds of formalizations by clearly describing which leadership tasks, through what fora and channels, are delegated to employees and by building an organizational structure facilitating employees having influence and participating in decision-making, in general, and regarding collectively issues. The two other cases (cases 2 and 3) had developed DL without including forms of formalization in their program theories concerning DL and were thus rather related to organizational culture and a common understanding of shared commitment and responsibility taking [44]. In the less formalized approaches to DL, the collaboration arising and the close relationship between managers and employees were rather based on a spontaneous organization as a kind of sensemaking [11] with roots in the values serving as the principal foundation of the workplace. We can see how the two different typologies of DL relate to Gronn’s [8] principles of DL, which, on the one hand, is an expression of institutionalization by formalizing relationships and activities to build collaborative structures into the organization and, on the other, an expression of spontaneous collaborations where individuals with different skills and expertise together contribute and thus gain responsibilities. The analysis of the study points out that it seems as if what contributes to experiences of DL is not the degree of formalization itself but rather how the approach to DL interacted with contextual factors. More specifically, the result indicated different degrees of formalization of organizational structures such as leadership training, coaching managers and team leaders, members representing self-organized groups, and other arenas and meeting venues to develop a basis for decision-making and make decisions collectively. In the most successful cases of DL, this has led to levels of stability in structures that enable and make DL sustainable over time. On the other hand, the results also highlight that too much formalization in an early stage of developing DL may constitute a hindering factor if employees are not mature/willing to take on delegated leadership tasks.

Concerning the mechanism of *participatory approaches to implementation* it shows that from the perspective of the support and participation of operational staff, the results point to the importance of a critical mass of employees being positive about the organization’s approaches and efforts to increase their taking on responsibilities and participating in decision-making. In this context the mechanism *participatory approaches to implementation* are critical. A more participatory and dialogue-based implementation approach seems to be a successful factor in combination with coaching employees in taking on responsibilities. A value and idea orientation regarding DL during implementation could also be stressed as a complement to be created simultaneously with building a formalization of DL. If no shared values and goals exist that govern work, it is also hard to take on responsibilities/engagement. The results are in line with extensive previous research highlighting the importance of participatory change management approaches, including clear visions for and support for change processes at different organizational levels (e.g.,[40, 41, 59]).

The importance of relational aspects of DL stands out including both the *mechanisms of vertical sense-making, and horizontal sense-making*. Agency over decision-making may be defined as a relational process cultivating employees to feel trusted to make decisions and to trust the decision-making of others (cp [7, 8]). Overall organizing for DL does not seem possible if trustful and good relations do not exist in the organization. Healthcare professionals are, together with the first-line managers, the professional group primarily subject to the various change processes. The results of this study highlight the importance of collaborative practices regarding DL and how communicative processes and decision-making may be put into practice when healthcare professionals work together. Marles [53] underlines the importance of collaborative practices contributing to common “sense-making.”

We have in this study assumed that greater alignment across organizational levels on views on DL will enable the mechanism of *vertical sense-making.* The different cases also show how critical vertical sense-making is for the development and sustainability of DL. The successful processes of DL in the studied cases were manifested by collaborative processes across organizational levels. In successful cases, there is overall strong support for consistently implementing DL, including collaborative processes across organizational levels. Furthermore, a high degree of engagement could be distinguished among politicians, upper management, first-line managers, and operational staff. Support and collaboration not only includes securing resources for developing DL but also having champions at different levels engaging in realizing the program theories.

In *the vertical sense-making processes* the results specifically point to the importance of first-line managers championing DL by coaching and motivating employees to gradually take on more responsibilities, thereby showing that they trust employees and working on reducing employee resistance. Coaching leadership may also be seen as in line with a learning approach, which may be highlighted as important for developing employees’ decision-making capacity [60]. Furthermore, connected to the mechanism of *horizontal sense-making* the most successful cases in this study had self-organized groups that developed a mutual understanding between first-line managers and employees in the organized groups of DL (cf [2]. The teams in this context focused on different aspects of learning to build a joint understanding of responsibility-taking and decision-making.

Summarizing the results, distributing leadership and developing a more trust-oriented organization to a great extent concerns giving health care professionals more room for maneuver and ultimately more power. Developing trust-based principles of governance such as DL may from a broader perspective be seen as efforts to find more sustainable ways of organizing eldercare. Here, intentions were observed to create more attractive work conditions as well as to promote efficiency and quality in work by giving operational staff greater decision authority about how to organize and perform work in line with the needs of clients [1]. However, current strained working conditions interact with the willingness of operational staff to take responsibility. For example, the results show that good working conditions represent an important factor in creating engagement for DL and sustaining it over time. In cases 4–6 where DL includes power over how to use resources the financial situation also needs to be stable to make it an attractive approach for employees. In the context of more strained financial conditions and with employees with less experience in management responsibilities, a more dialogue-orientated approach, such as in case 1, may also be seen as a suitable first approach to DL, as it focuses on the process of building cooperation and shared responsibilities. Strained conditions, including high sick leave and high staff turnover rates, may furthermore hinder processes of developing shared visions, shared understandings, etc., which are necessary for collaborative decision-making processes.

Through a systematic analysis of generative mechanisms, we have tried in this article to highlight what makes things happen or not happen in the various cases we investigated. As we described in the method section, we have through retroduction argued our way to understand how and why DL is implemented in different contextual environments. The approach has given us a clear picture of the basic conditions that should exist for DL to be implemented, and it has also led to the conclusion that the most basic generative mechanisms are what we describe in the article as "sense-making" in the sense of a basic horizontal and vertical common understanding around goals and meaning of DL. The results of the study are limited by mirroring six specific cases, of which three belong to the same municipality sector. Including more cases in a more quantitative-oriented study could be recommended for analyzing the proposed mechanisms of this study further.

## Conclusions

In the study, we see examples of the different aspects of DL highlighted by Gronn [8]. As Gronn puts it, this may involve *spontaneous collaborations,* where individuals with different skills and expertise contribute and thus gain responsibilities. It may concern an *intuitive understanding* based on a professional working relationship, and it may involve an *institutionalization* of these relationships and activities to build collaborative structures in the organization. These are important learnings for eldercare in general. It both highlights and emphasizes the importance of prioritizing aspects related to team collaboration and building of relationships.

Regardless of the path toward DL chosen by the various eldercare workplaces in this study, the common denominator for those having succeeded in distributing leadership is developing a relational agency based on shared visions, a shared understanding of roles and responsibilities, a learning approach, and a dialogue-oriented relationship between management and employees. The mechanisms of participatory approaches to implementation, vertical sense-making, and horizontal sense-making contributed can thus be seen as the most critical mechanisms for the development of DL. An additional key factor is experiencing having sufficient resources, thereby making it attractive to take on increased responsibilities. The mechanisms of formalization (i.e., when trust-based structures are built into the organization) naturally mean an increased ability to maintain DL as a leading principle over time. A high degree of involvement may, however, emerge from a value and idea orientation approach without the degree of formalization being particularly high.

Practical implications from the studies are that DL can be designed in several different ways depending on prerequisites and contextual conditions. A formalization of trust-oriented structures is a measure that stabilizes DL, but the central aspect concerns horizontal involvement that includes the entire staff as well as vertical involvement that extends across different system levels in the organization. These findings indicate that when you want to introduce DL, preparatory work is required which means that all concerned are involved and understand both the purpose and goal of the change, and that this purpose and goal is backed up by management at higher levels. The following concrete advice is hereby provided to work organizations for promoting the mechanisms related to development of DL:

- First and foremost, it is essential to establish work structures that promote a dialogue-oriented involvement of staff and first-line managers for promoting participatory approaches to the implementation of DL.
- Secondly, it is crucial to develop formalization in form of structures (e.g. meetings, routines, and forums) that bring relational sustainability, ensuring the continued involvement of staff and managers.
- Thirdly, it is necessary to create conditions through forums for dialogue within teams and between key actors at different organizational levels that facilitate continuous work on vertical and horizontal sense-making to establish consensus on the content, objectives and visions of DL.

This study has been limited to analyze DL within eldercare in Swedish municipalities. Further studies are recommended to investigate the mechanisms of DL within eldercare in other national settings as well as to analyze associations between the mechanisms of formalization processes, participatory approaches to implementation, vertical sense-making, and horizontal sense-making and DL in quantitative data material.

## Supplementary Information

Supplementary Material 1.

Supplementary Material 2.

## Data Availability

The datasets generated and/or analyzed during the current study are not publicly available as this would compromise the privacy of individual participants who partook in the interviews and observations but are available from the corresponding author upon reasonable request.

## Abbreviation

DL: Distributed leadership
