# Supplementary material for: Forms of distributed leadership – a case study of six workplaces in eldercare
Source: BMC Health Serv Res. 2025 Feb 22;25:300. doi: 10.1186/s12913-025-12417-1 (PMC11847337; doi:10.1186/s12913-025-12417-1)
Supplement: Supplementary file 1 — Supplementary Material 1. [file 12913_2025_12417_MOESM1_ESM.docx]

# COREQ-Report

| Domain and chechpoints | Line number |
| --- | --- |
| Domain 1: Research team and reflexivity |  |
| 1. Information about interviewer/facilitator: Which author/s conducted the interview or focus group? | 362-364 |
| 2. Information about credentials: What were the researcher’s credentials? E.g. PhD, MD | 324 |
| 3. Information about occupation: What was their occupation at the time of the study? | 325 |
| 4. Information about gender: Was the researcher male or female? | 364 |
| 5. Information about experience and training: What experience or training did the researcher have? | 322-325 |
| **Relationship with participants** |  |
| 6. Information about relationship established: Was a relationship established prior to study commencement? | 334-336 |
| 7. Information about participant knowledge of the interviewer: What did the participants know about the researcher? e.g. personal goals, reasons for doing the research | 346-347 |
| 8. Information about interviewer characteristics: What characteristics were reported about the interviewer/facilitator? e.g. Bias, assumptions, reasons and interests in the research topic. | 346-347 |
| Domain 2: study design |  |
| **Theoretical framework** |  |
| 9. Information about methodological orientation and Theory: What methodological orientation was stated to underpin the study? e.g. grounded theory, discourse analysis, ethnography, phenomenology, content analysis | 327 |
| **Participant selection** |  |
| 10. Information about sampling: How were participants selected? e.g. purposive, convenience, consecutive, snowball | 341-342 |
| 11. Information about method of approach: How were participants approached? e.g. face-to-face, telephone, mail, email | 347-357 |
| 12. Information about sample size: How many participants were in the study? | 359 |
| 13. Information about non-participation: How many people refused to participate or dropped out? Reasons? | 364-365 |
| **Setting** |  |
| 14. Information about setting of data collection: Where was the data collected? e.g. home, clinic, workplace | 374-377 |
| 15. Information about presence of non-participants: Was anyone else present besides the participants and researchers? | 378 |
| 16. Description of sample: What are the important characteristics of the sample? e.g. demographic data, date | 330-344,1425 |
| **Data collection** |  |
| 17. Information about interview guide: Were questions, prompts, guides provided by the authors? Was it pilot tested? | 384-396 |
| 18. Information about repeat interviews: Were repeat interviews carried out? If yes, how many? | 400 |
| 19. Information about audio/visual recording: Did the research use audio or visual recording to collect the data? | 398 |
| 20. Information about field notes: Were field notes made during and/or after the interview or focus group? | 405-406 |
| 21. Information about duration: What was the duration of the interviews or focus group? | 381-382 |
| 22. Information about data saturation: Was data saturation discussed? | 465-466 |
| 23. Information about transcripts returned: Were transcripts returned to participants for comment and/or correction? | 398-400 |
| Domain 3: analysis and findings |  |
| **Data analysis** |  |
| 24. Information about number of data coders: How many data coders coded the data? | 464-465 |
| 25. Information about description of the coding tree: Did authors provide a description of the coding tree? | 415-462 |
| 26. Information about derivation of themes: Were themes identified in advance or derived from the data? | 415-462 |
| 27. Information about software: What software, if applicable, was used to manage the data? | 461 |
| 28. Information about participant checking: Did participants provide feedback on the findings? | 461-462 |
| **Reporting** |  |
| 29. Information about quotations presented: Were participant quotations presented to illustrate the themes / findings? Was each quotation identified? e.g. participant number | 460-461 |
| 30. Information about data and findings consistent: Was there consistency between the data presented and the findings? | 460-461 |
| 31. Information about clarity of major themes: Were major themes clearly presented in the findings? | 457-458 |
| 32. Information about clarity of minor themes: Is there a description of diverse cases or discussion of minor themes? | 457-458 |
