# Supplementary material for: Forms of distributed leadership – a case study of six workplaces in eldercare
Source: BMC Health Serv Res. 2025 Feb 22;25:300. doi: 10.1186/s12913-025-12417-1 (PMC11847337; doi:10.1186/s12913-025-12417-1)
Supplement: Supplementary file 2 — Supplementary Material 2. [file 12913_2025_12417_MOESM2_ESM.docx]

# Appendix 1 Thematic topic guide, interviews

1. Topic guide managers

**Background**

- Description of background including experiences of being a manager
- How would you describe your work situation and position in the organization?

**Topic 1. Programme theory**

- What change measures have or are planned to be implemented? (What is planned to be done)
- Why do you think these change measures are needed? (The motives for measures)
- What results do you expect? (Effects)

**Topic 2. Experiences of implementation**

- Has your leadership been affected or changed in connection with the change measures you have implemented (Degree of Distributed Leadership)
- How is development work followed up and evaluated?
  - What kind of feedback is given?
  - What results are requested?
- How have the changes you have implemented impacted the role of the employees?
  - Positive effects?
  - Challenges?
- How is the work of the management team functioning?
  - What is their work and their leadership characterized by?
- How do you look upon the employees' influence and co-determination? (Degree of Trust Organization)
- Based on an ideal situation: What does trust between you and your employees mean to you?
  - How does it look today?
  - Can you give examples of differences in trust compared to before implementation of changes?
- What have the most important learnings been?
  - What questions do you have continuous discussions on?
  - What are you most content with?

**Topic 3. Pre-conditions, conflicts and hindering factors**

- Has there been any challenges during the change of work? (Conflicts)
- What obstacles or difficulties have arisen during the change of work?
  - Which obstacles in the organization do you think were the most difficult to master? (hindering mechanisms)
- Is the whole organization aware of the change measures? (Relationships between system levels)
- Which communication channels, formal and informal, are important for your work?
- How would you like to describe your organizational culture?
  - What norms, values and expressions of community are important to you?
- Is the entire organization aware of the current change measures? (Relation between system levels)
- What do the organizational conditions look like for the change of work that is now being carried out?
- In which forums/situations does communication/learning take place?
- What does trust, dialogue and communication look like between a) employees and closest management; b) employees – senior management; c) closest management-senior management.
  - What do learning processes look like between the levels?

1. Topic guide employees

**Background**

- Education,
- Time of employment at the workplace
- Previous work experience

**Topic 1. Experiences of change measures**

- What do you think about the change of work?
  - Positive aspects?
  - Negative aspects?
- Do you experience differences in influence/responsibility/co-determination?
- Have you been involved in or otherwise noticed the ongoing change measures?
- What is working well?
  - What could be improved?
- What is the most critical factor for success of the change of work?
- Would you recommend the change measures to other workplaces?

**Topic 2 Co-determination and distributed leadership.**

- Do you consider yourself involved in the decisions that are made at your workplace?
- Are there issues at your workplace that you would like to be involved in and influence to a greater extent?
- Describe the role of your closest manager.
- Describe how you work in your work team.
- What areas are you responsible for?

**Topic 3. Relationships, trust, and job satisfaction**

- How is it to work at the workplace?
  - What is working well?
  - What could be improved?
- Are you satisfied at work?
  - With what aspects?
  - What are you less satisfied with?
- To what extent do you experience trust?
  - Trust in you from managers?
  - Do you trust managers and colleagues?
  - Trust within the work team?
- Do you look forward to further development of the change measures?
  - Do you see any future risks with the change measures?
